# Widespread, Reversible Cysteine Modification by Methylglyoxal Regulates Metabolic Enzyme Function

**DOI:** 10.1021/acschembio.2c00727

**Published:** 2022-12-23

**Authors:** John S. Coukos, Chris W. Lee, Kavya S. Pillai, Kimberly J. Liu, Raymond E. Moellering

**Affiliations:** Department of Chemistry, The University of Chicago, 929 E. 57th Street, Chicago, Illinois 60637, United States

## Abstract

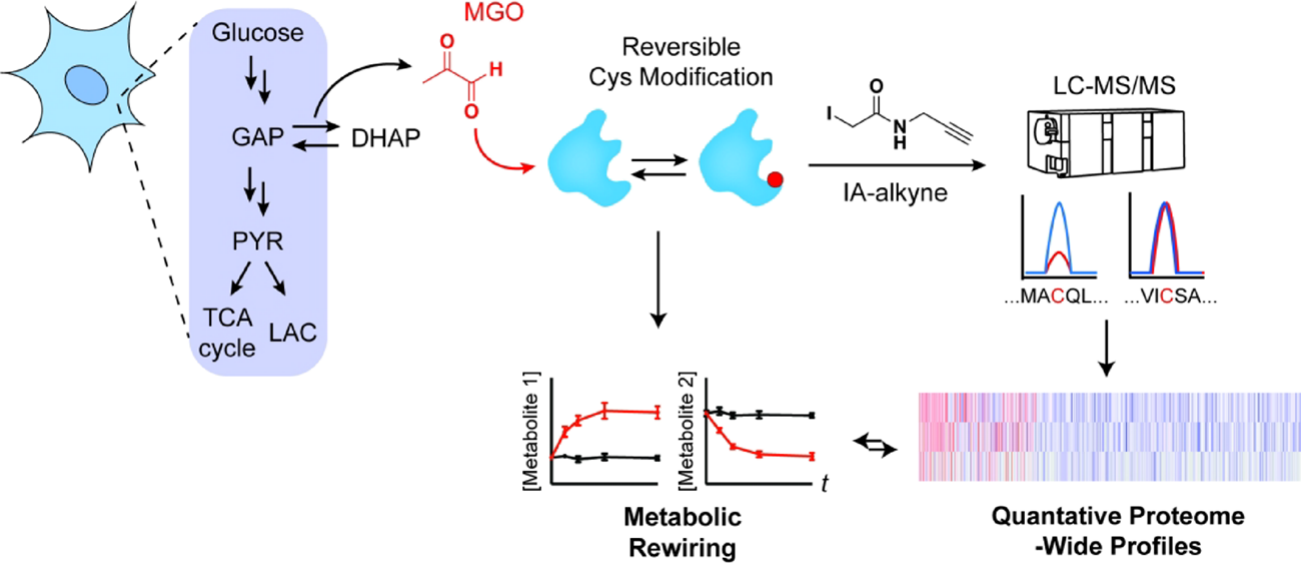

Methylglyoxal (MGO), a reactive metabolite byproduct of glucose metabolism, is known to form a variety of posttranslational modifications (PTMs) on nucleophilic amino acids. For example, cysteine, the most nucleophilic proteinogenic amino acid, forms reversible hemithioacetal and stable mercaptomethylimidazole adducts with MGO. The high reactivity of cysteine toward MGO and the rate of formation of such modifications provide the opportunity for mechanisms by which proteins and pathways might rapidly sense and respond to alterations in levels of MGO. This indirect measure of alterations in glycolytic flux would thereby allow disparate cellular processes to dynamically respond to changes in nutrient availability and utilization. Here we report the use of quantitative LC–MS/MS-based chemoproteomic profiling approaches with a cysteine-reactive probe to map the proteome-wide landscape of MGO modification of cysteine residues. This approach led to the identification of many sites of potential functional regulation by MGO. We further characterized the role that such modifications have in a catalytic cysteine residue in a key metabolic enzyme and the resulting effects on cellular metabolism.

## Introduction

Methylglyoxal (MGO) is a reactive α-oxoaldehyde byproduct of several metabolic processes with glycolysis being a dominant source of production. Due to the ubiquitous and reactive nature of MGO, it has been shown to participate in nonenzymatic chemical reactions with proteins, metabolites, and nucleic acids.^1−8^ Moreover, the presence of two reactive carbonyl groups within this three-carbon metabolite enables myriad inter- and intramolecular crosslinking reactions to occur within and between these biomolecules.^9−11^ The vast majority of published studies on MGO-mediated posttranslational modifications (PTMs) have focused on relatively few species, such as hydroimidazolone (MG-H1) lesions on protein arginines, likely due to their abundance, stability, and availability of analytical tools (e.g., polyclonal antibodies). Work with purified proteins and more recently in native proteomes have shown that MGO can form reversible hemithioacetal modifications^12^ and stable mercaptomethylimidazole (MICA) modifications^13^ on protein cysteines. Cysteines are the most nucleophilic of the proteinogenic amino acids and, as a result, play prominent functional roles in many different classes of proteins, including as catalytic centers, redox-active sensors, and mediators of dynamic protein interaction surfaces.^14,15^ Recently, we demonstrated in a complex mix of nucleophiles how this nucleophilicity makes adduction of MGO to thiols such as cysteine or glutathione the kinetically favored reaction.^4^ This phenomenon likely mirrors the cellular environment where MGO may form reversible MGO adducts with glutathione or reactive cysteine residues on proteins. In fact, it is estimated based on both modeling and experimental evidence that only a small percentage of intracellular MGO—perhaps as little as 1%—exists as free MGO at any given moment.^12,16^ The rest is reversibly bound to biomolecules likely in the form of hemithioacetals.

Given this reactivity landscape, we have hypothesized that, especially under conditions of high glycolytic flux, there may be an appreciable amount of MGO engaged in reversible modifications on a number of the more reactive or accessible cysteine residues throughout the cell. However, to the best of our knowledge, only a couple of examples of functional MGO-derived cysteine modifications have been characterized at the molecular level most notably the Cys-to-Arg MICA crosslink in the redox sensor protein KEAP1.^13^ This crosslink involves a critical sensor cysteine (C151) of KEAP1 and impairs KEAP1-dependent ubiquitylation and degradation of the transcription factor NRF2. This signaling mechanism thereby links glucose metabolism and MGO stress to a feedback response activation of critical antioxidant genes. More recently, MICA crosslinking of tetramers of metabolic enzyme IMPDH2 was demonstrated.^17^ The modifications, which occur between the catalytic C331 residue and one of several arginine residues, were shown to regulate enzymatic activity of IMPDH2 in vitro though further studies exploring the cellular effects of this mechanism are needed. Based on identification of stable inter-residue MGO modifications on proteins like KEAP1 and IMPDH2, as well as kinetically controlled engagement of protein- and metabolite thiols, we posited that there likely are other examples of functional stable or reversible MGO-derived cysteine modifications throughout the proteome. Identification of these labile chemical events in native environments like whole proteome or live cells, however, remains a significant challenge in the field of proteomics. Proteome-wide, quantitative profiling of MGO-derived cysteine modifications is virtually impossible using traditional LC–MS/MS-based proteomic approaches. No antibodies or other techniques currently exist for enrichment of hemithioacetal or MICA modifications. Additionally, the distinct chemical natures of these modifications may pose challenges to production of pan-specific antibodies. Most importantly, MGO-dependent hemithioacetals are fairly labile and would most likely not survive enrichment or sample processing steps intact.

Efforts to systematically identify MGO modifications at specific sites within proteins have predominately relied on the use of nonnatural MGO surrogates, like alkynylated dicarbonyl probes.^18,19^ While these studies have identified numerous sites of MGO modification, neither is capable of reporting on reversible modifications at nucleophilic residues like cysteine. Therefore, we sought to apply a competitive chemoproteomic profiling approach to detect, quantify, and prioritize potential sites of reversible or irreversible modification with cysteines in cancer cell proteomes.

## Results and Discussion

### Proteome-Wide Detection of MGO-Mediated Cysteine Engagement

**Figure 1:**
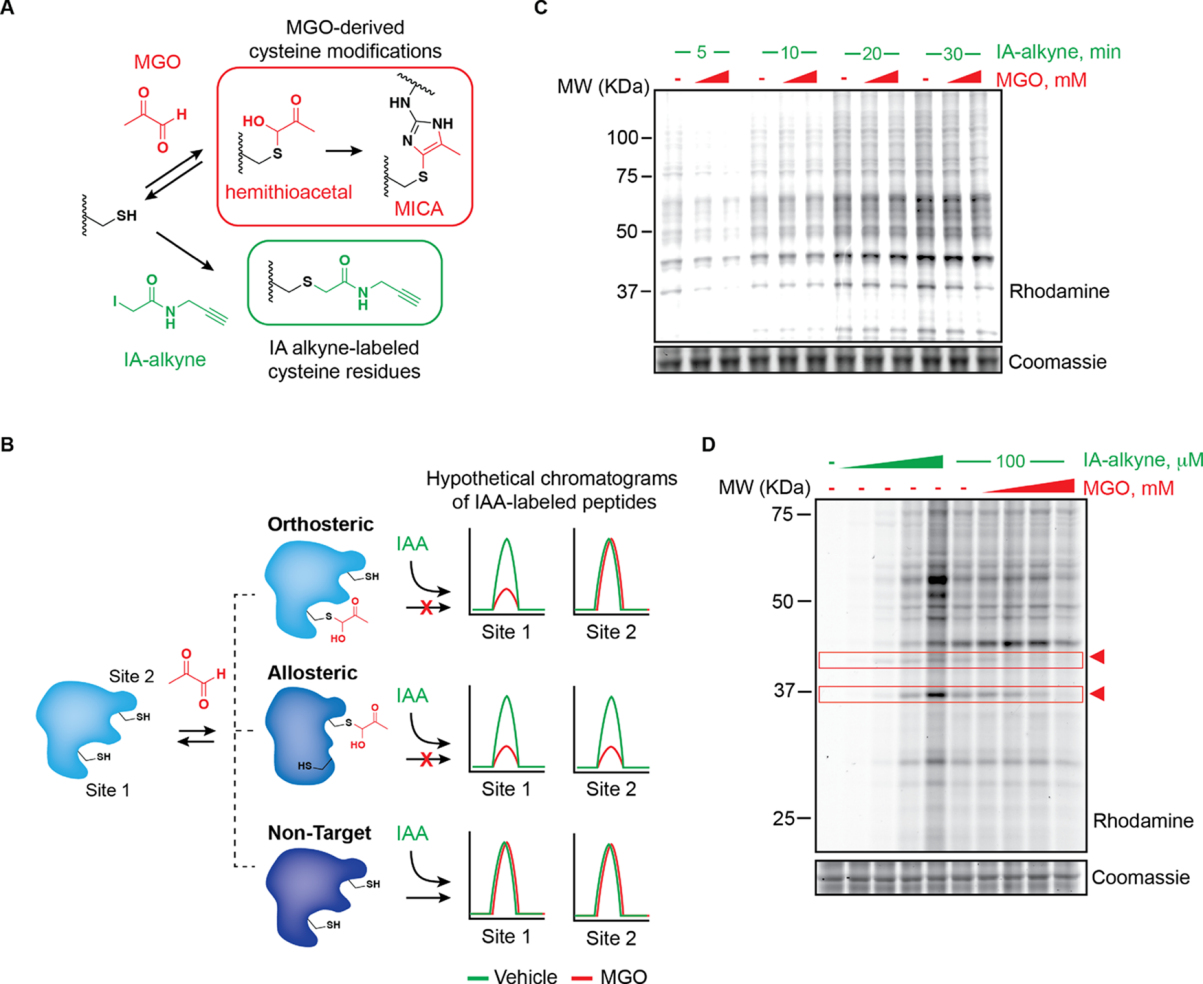
Chemoproteomic detection of MGO modifications on protein cysteines. (A) Indirect cysteine reactivity profiling with iodoacetamide alkyne (IA-alkyne). Methylglyoxal (MGO)-modified cysteines, i.e., reversible hemithioacetal or stable MICA modifications, do not engage with the IA-alkyne probe. (B) Schematic depicting the possible modes of MGO-regulated cysteine engagement with the IA-alkyne probe. (C) Fluorescent gel electrophoresis of HeLa lysates pretreated with 0.25 or 1 mM MGO for 1 h followed by IA-alkyne probe (100 μM) treatment for the indicated time points. (D) Fluorescent gel electrophoresis of HeLa lysates treated with IA-alkyne probe (0–200 μM) for 1 h (left) or pretreated with MGO (0–1 mM) followed by IA-alkyne (100 μM, 1 h; right). Arrowheads highlight bands competed by MGO in a dose-dependent manner. Lysates in C and D were labeled with rhodamine azide for in-gel fluorescence visualization. Coomassie staining was performed as a loading control.

Although MGO is known to modify cysteine residues either through reversible hemithioacetals or through stable MICA modifications, both are challenging to identify via traditional proteomic profiling. To identify sites of MGO modification proteome-wide, we employed a competitive reactivity profiling approach using the cysteine specific probe iodoacetamide alkyne (IA-alkyne). IA-alkyne covalently modifies free reactive cysteine residues via nucleophilic displacement of the iodine by the cysteine side chain thiolate. The resulting alkylated cysteine is chemically stable, enabling bio-orthogonal labeling of the pendant alkyne with a fluorophore for intact protein visualization by gel electrophoresis, or labeling with retrieval moieties like biotin or desthiobiotin for protein- or peptide-level enrichment and analysis by LC–MS/MS proteomics. In principle, covalent interactions between protein cysteines and MGO should be detectable so long as the residence time significantly prevents covalent labeling by IA-alkyne (Figure 1A). We further reasoned that three scenarios could be detected using this approach: (1) significant occlusion of IA-alkyne labeling of a specific cysteine caused by direct MGO modification in lysates or cells, (2) altered IA-alkyne labeling of one or more cysteines due to MGO engagement at distal, allosteric sites on the target protein, and (3) negligible or suitably short-lived MGO engagement of specific protein cysteines, resulting in no significant effect on IA-alkyne engagement (Figure 1B). We first tested whether acute MGO treatment of whole, homogenized HeLa proteome appreciably altered IA-alkyne labeling as measured by fluorescence gel electrophoresis following [3+2] Huisgen “click” labeling of alkyne-labeled proteins with rhodamine azide (Figure S1). Intriguingly, MGO treatment followed by a short incubation with IA-alkyne resulted in significant, dose-dependent reduction of IA-labeled protein cysteines (Figure 1C). Extended treatment with IA-alkyne, on the other hand, largely alleviated MGO competition, suggesting that while many accessible cysteine residues are capable of forming reversible (e.g., hemithioacetal) modifications with MGO, the majority of these sites are in rapid equilibrium and will ultimately be outcompeted by the irreversible IA-alkyne modification at longer timepoints (Figure 1C). Competitive treatment of HeLa lysates with an intermediate concentration of IA-alkyne after pretreatment with MGO demonstrated that even using relatively long IA-alkyne incubations (1 h), there were several apparent bands competed by MGO, including some with different dose–response profiles (Figure 1D). These data suggested that a subset of protein cysteines may be either irreversibly modified by MGO or engaged in kinetically trapped reversible modifications resulting in longer residence times and, by extension, increased potential for functional effects on those proteins and pathways.

**Figure 2:**
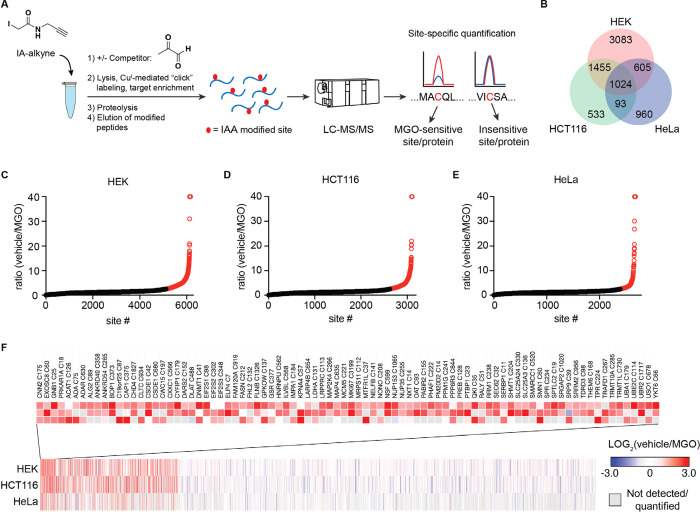
Proteome-wide profiling of MGO-IA-alkyne competition at functional cysteines. (A) Workflow of IA-alkyne SILAC LC–MS/MS profiling experiments to quantify MGO regulation of cysteine residues in a proteome-wide manner. (B) Venn diagram of unique, quantified cysteine residues in lysates from HEK293, HCT116, and HeLa cancer cells. (C–E) Waterfall plots of IA-alkyne-labeled cysteine residue SILAC ratios for HEK293 (C), HCT116 (D), and HeLa (E) lysates treated for 2 h with 1 mM MGO or vehicle at 37 °C. Data points shown are mean SILAC ratio derived from *n* = 4 biological replicates each. (F) Heatmap of the ratios of all unique sites quantified in lysates from multiple cell lines in IA-alkyne SILAC proteomic profiling experiments. Sites that showed a SILAC ratio of vehicle over MGO treated >2.5 in more than one cell line are highlighted. Gray boxes denote no data for that site/condition pair.

To generate a proteome-wide, quantitative, and site-specific profile of MGO-modified cysteine residues, we adapted a SILAC-based competitive IA-alkyne profiling workflow (Figure 2A).^14,20,21^ Pairs of isotopically “heavy” and “light” lysates from multiple cell lines were pulse-treated with MGO or vehicle followed by an IA-alkyne chase treatment. Subsequent click chemistry mediated labeling of alkyne-labeled proteins with a cleavable diazo biotin azide linker permitted selective capture and release of peptides modified by the IA-alkyne probe (Figure S1). Following enrichment on streptavidin-coated agarose beads, probe-labeled peptides were eluted and analyzed by LC–MS/MS proteomics. In this workflow, the SILAC ratio of the untreated versus treated samples for each cysteine residue yields an estimated percent occupancy of MGO. Measurements of free MGO have typically shown it in the low micromolar range; however, the vast majority of cellular MGO is reversibly bound to biomolecular nucleophiles such as cysteine residues.^12^ Approaches that have accounted for this reversibly bound pool of MGO indicate a true cellular concentration potentially as high as 300 μM.^16^ Given these wide estimates of cellular MGO concentration, as well as the clear propensity for the irreversible IA-alkyne labeling event to outcompete a potentially reversible MGO modification, balanced with the inherent need for high IA-alkyne labeling efficiency for target cysteine detection and quantification, we used a relatively high concentration of MGO (1 mM) for competitions with the goal of identifying the most highly altered cysteines in several cell lines. Using this approach, we quantified the extent of MGO modification of 7752 unique cysteine residues in treated lysates from HEK293, HeLa, and HCT116 cancer cells (Figure 2B, Datasets 1–3). We categorized cysteines as appreciably affected by MGO as having greater than a 2.5-fold MGO-to-vehicle SILAC ratio in two or more cell lines (Figure 2C–F); the vast majority of detected cysteines were not appreciably competed by MGO. Among the 3176 sites that were quantified in multiple cell lines and more than 1000 quantified in all three cell lines, only 86 cysteines (2.7%) were significantly competed under these conditions (Figure 2F). Within this group, there were notable cysteines present in diverse protein families from distinct subcellular locations and organelles and in a wide range of absolute abundance. Significant competition was observed at cysteines in several metabolic enzymes, including lactate dehydrogenase (LDHA) C131, cytosolic serine hydroxymethyltransferase (SHMT1) C204, acetoacetyl coenzyme A (CoA) acetyltransferase 1 (ACAT1) C126, and others. Additionally, there were many MGO-regulated sites observed on enzymes involved in regulation of PTMs and nucleotide modification, including ubiquitin-conjugating enzyme E2 C (UBE2C) C114, serine palmitoyltransferase 2 (SPTLC2) C19, protein phosphatase 1G (PPM1G) C241, DNA (cytosine-5)-methyltransferase 1 (DNMT1) C41, and others. Structural analysis of identified cysteines revealed a number of these sites for which modification would be predicted to directly inhibit enzymatic function, including the catalytic C126 residue of ACAT1, the active site C204 residue of SHMT1 for which covalent modification has been shown to inhibit protein function,^22^ and the catalytic C114 residue of ubiquitin-conjugating enzyme UBE2C.

Notably absent in all of these data sets was C151 of KEAP1, which we and others have previously shown to interact with MGO and other electrophiles.^13,23^ Several other cysteine residues from KEAP1, including C288, C297, and C319, were detected and quantified each in a single cell line with C319 being highly competed with a ratio of 40 in HEK293 cells. Despite its well characterized nucleophilicity, C151 is not detected in many previous proteomic profiling studies,^14,21,24^ KEAP1 is a low abundance protein,^25^ and C151 is particularly sensitive to oxidation, which would also block labeling by IA-alkyne. This suggests that there are likely other MGO-regulated cysteines that were not amenable to profiling by this method due to low abundance, poor tryptic peptide characteristics, or particular sensitivity of the cysteine to oxidation, which would be exacerbated upon cell lysis. For example, the active site C53/C52 of peroxiredoxin proteins PRDX1/2, which are transiently oxidized to sulfenic acid during the protein catalytic cycle, is also not detected. Conversely, the resolving cysteine residues C172/C171 of PRDX1/2 are both detected and not seen to be MGO-regulated in these data sets.

Motif analysis of sites that showed a competition ratio of 2.5 or greater in more than one cell line revealed no strong trends, suggesting that the primary sequence surrounding the cysteine residues did not play a significant role in determining their reactivity toward methylglyoxal (Figure S2A). Conversely, secondary structure analysis of the sequences within which the cysteine residues were contained showed enrichment in unstructured loop regions around top competed sites as compared to all sites that were quantified in more than one cell line (Figure S2B). This trend correlated with the observation that top competed cysteine residues were more likely to be solvent-exposed and in disordered regions of proteins compared to all cysteines profiled (Figure S2C).

### MGO-Dependent Regulation of Functional Cysteines in Metabolic Enzymes

**Figure 3:**
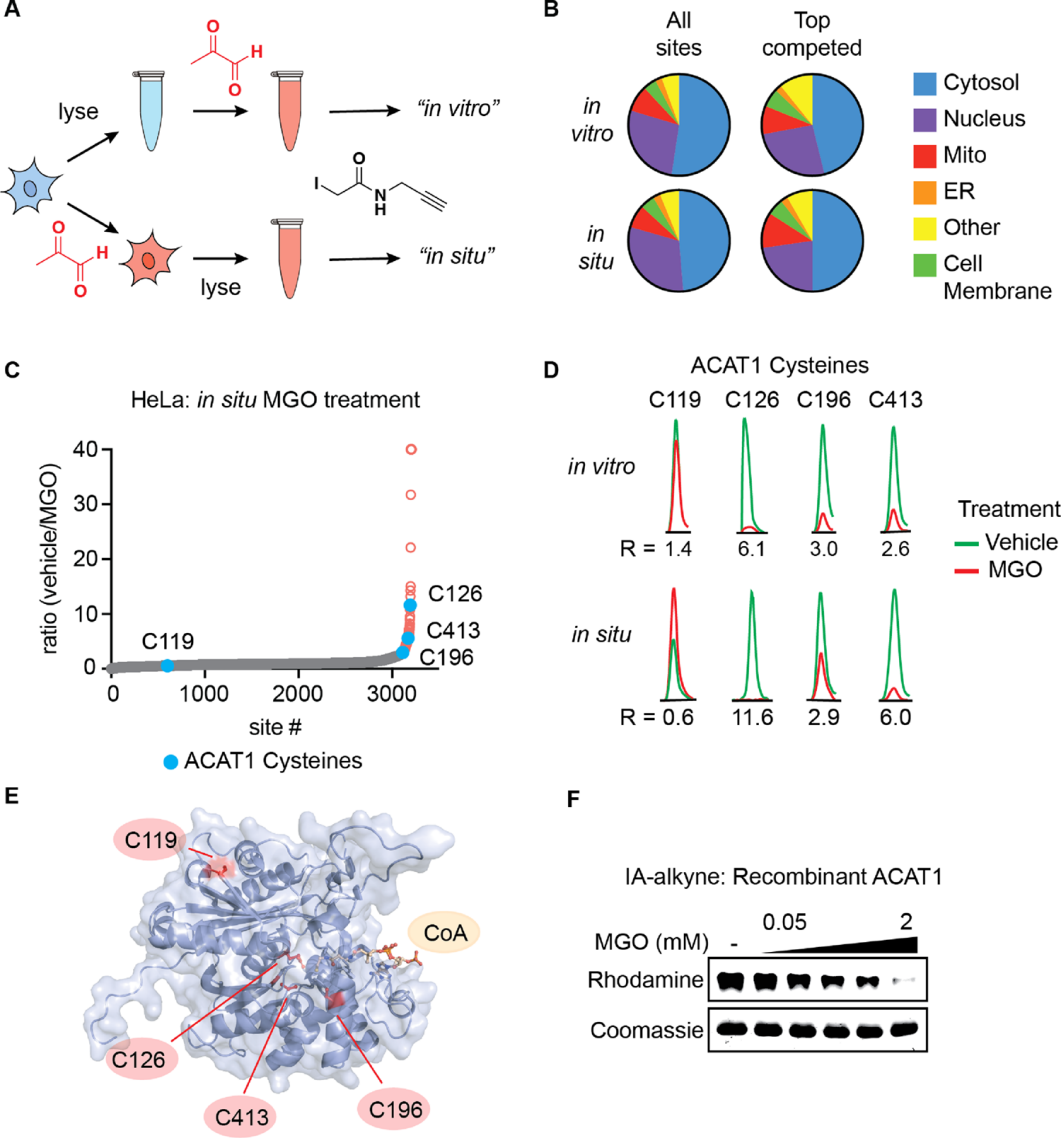
MGO modifies active site cysteine residues of metabolic enzyme ACAT1. (A) Schematic depicting comparative MGO treatment workflow for “in vitro” vs “in situ” proteomics samples. (B) Distribution of all cysteines and top competed cysteine residues with a ratio >2.5 in IA-alkyne SILAC experiments with HeLa lysate (“in vitro”) and HeLa cells (“in situ”) treated with MGO or vehicle across proteins localized to the indicated subcellular compartments. (C) Waterfall plot graphs of IA-alkyne-labeled cysteine residue SILAC ratios for HeLa cells treated for 2 h with 2 mM MGO or vehicle at 37 °C with cysteine residues from ACAT1 highlighted. (D) Representative chromatograms of labeled peptides of ACAT1 from IA-alkyne SILAC experiments with HeLa lysate and HeLa cells treated with MGO or vehicle. (E) Structure of ACAT1 active site, depicting acetylated C126 with cysteine residues quantified in IA-alkyne proteomics experiments highlighted (PDB accession: 2F2S). (F) Dose-dependent competition of ACAT1 and IA-alkyne by MGO in vitro. Recombinant ACAT1 (0.05 mg/mL) was pretreated with indicated concentrations of MGO for 2 h followed by IA-alkyne treatment for 30 min.

We next sought to interrogate MGO engagement of cysteines in live cells. We reasoned that the combination of reversible covalent modification and extended processing time to lyse and chase with the irreversible IA-alkyne probe may only capture the most stable, affected sites in the proteome. We also aimed to capture direct MGO interactions with protein cysteines, rather than downstream effects due to extended changes in the redox environment and/or signaling.^13,21^ To identify relevant time courses for formation of protein modifications in cells, we measured the kinetics of MGO-mediated modification of cellular glutathione in cells. Treatment of live HeLa cells with MGO resulted in rapid accumulation of lactoylglutathione and the MICA crosslink between arginine and glutathione, which peaked within approximately 2 h (Figure S3 and Table S1). In total, reduced glutathione and free arginine levels were not significantly affected across these and other timepoints out to 8 h (Figure S3). Taken together, these results suggested a 2-h treatment time for cell-based treatment and subsequent proteomic profiling would be optimal for monitoring formation of MGO modifications on cysteine residues. Similar to other studies in the literature, we exposed live HeLa cells to a high-dose pulse of MGO to account for the low efficiency of MGO exposure in the cell^13,18,26,27^ and to maintain high-occupancy of relevant cysteines that will endure cellular lysis, IA-alkyne chase, and subsequent proteomic processing for LC–MS/MS profiling (Figure 3A–C). We reproducibly detected and quantified SILAC competition ratios from 3206 unique protein cysteines, among which a significantly smaller fraction (∼4%) was significantly competed relative to our lysate-based profiling experiments (Figure 2B–E, Dataset 4). This was expected given the likelihood of reversible modifications being lost during the extended processing time.

Broad comparison of all detected cysteines and those that were significantly competed by MGO showed essentially identical subcellular localization distribution in both our in vitro and in situ profiles from HeLa cells with cytosolic proteins representing ∼50% in each group (Figure 3B). As with the in vitro profile, top competed cysteine residues in situ with a ratio greater than 2.5 were more likely to be present in solvent-exposed and disordered regions of proteins than all cysteines profiled (Figure S4A,B). Whereas the most competed cysteines in vitro were enriched in unstructured loop regions, this trend was less defined in the more restricted in situ data set (Figure S4C).

**Figure 4:**
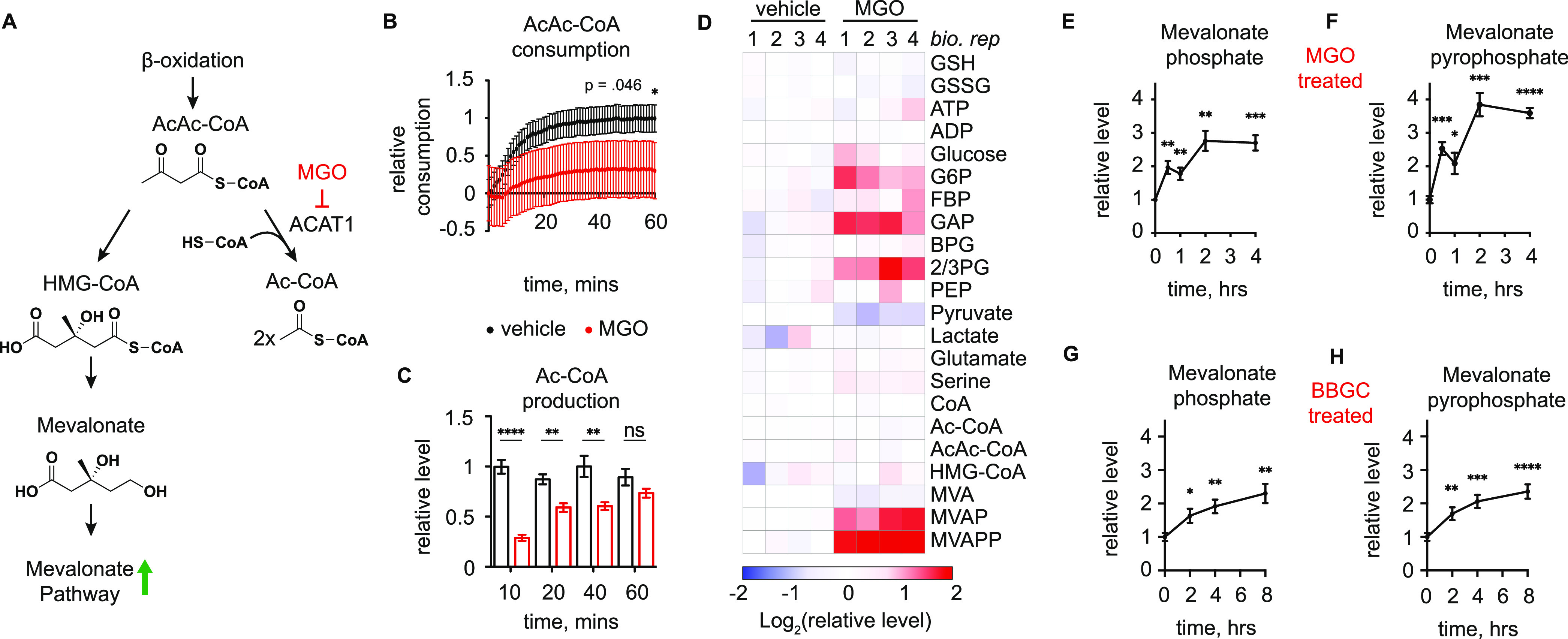
Methylglyoxal modification regulates ACAT1 activity. (A) Connections between ACAT1 and mevalonate pathway, as well as a proposed model of mevalonate pathway metabolite accumulation as a result of MGO inhibition of ACAT1 activity. (B) Relative consumption of AcAc-CoA by recombinant ACAT1 treated with 2 mM MGO (red) or vehicle (black). (C) LC–MS quantification of Ac-CoA produced by recombinant ACAT1 within time course studies after pretreatment with 2 mM MGO or vehicle. (D) LC–MS quantification of indicated metabolites in HeLa cells treated with MGO (2 mM) or vehicle for 4 h. (E,F) LC–MS quantification of mevalonate-5-phosphate (E) and mevalonate-5-pyrophosphate (F) in HeLa cells treated with MGO (2 mM) at the indicated time points. (G,H) LC–MS quantification of mevalonate-5-phosphate (G) and mevalonate-5-pyrophosphate (H) in HeLa cells treated with GLO1 inhibitor BBGC (20 μM) at the indicated time points. Data are mean ± S.E.M. from *n* = 8 (B,G,H) or 4 (C,E,F) independent biological replicates. Statistical analysis in B and C is by one-sided unpaired Student’s *t*-test. Statistical analysis in E–H is by two-sided unpaired Student’s *t*-test comparing individual timepoints to their corresponding 0-h control. *p < 0.05; **p < 0.01; ***p < 0.001; and ****p < 0.0001.

Analysis of in vitro and in situ data sets identified some protein cysteines that were consistently and significantly competed by MGO, potentially representing more stable and functionally relevant sites of modification (Figure 3D). Near the top of this list was the metabolic protein acetyl-coenzyme A acetyltransferase ACAT1 (not to be confused with acyl-coenzyme A: cholesterol acyltransferase also known as SOAT1), which contains IA-alkyne-labeled cysteines within and adjacent to its active site (Figure 3E). The active site residue C126 is known to perform a trans-thioesterification with its substrate AcAc-CoA, resulting in an enzyme-bound acetyl-thioester. C126 was among the top MGO-competed residues in both in vitro and in situ data sets (Figures 2F and 3C,D). Two proximal cysteines in the ACAT1 catalytic center, C196 and C413, were also significantly competed by MGO. By contrast a solvent-exposed cysteine, C119, was detected but not competed in either in vitro or in situ data sets (Figure 3C–E), confirming that the high-degree of competition of active site residues is specific to those sites and not a general phenomenon. Pulse-chase treatment of recombinant ACAT1 with MGO and IA-alkyne followed by gel-based visualization confirmed appreciable and dose-dependent competition of cysteines by MGO (Figure 3F). The partial reduction of aggregate, IA-alkyne-labeled ACAT1 signal at low doses suggests that moderate local concentrations of MGO in cells may reversibly modify C126 in cells. Given the lack of proximal arginine residues capable of forming a MICA crosslink in the active site of ACAT1, we speculated that the MGO modification of the active site cysteines was likely a reversible, hemithioacetal modification. To interrogate this reversibility, we incubated recombinant ACAT1 with a MGO and IA-alkyne pulse-chase approach and compared the effect of a buffer-exchange wash out step after the MGO treatment. We observed recovery of IA-alkyne labeling post-wash-out, which supports the reversibility of this modification at active site cysteines in ACAT1 (Figure S5). Finally, we confirmed that MGO inhibited the consumption of AcAc-CoA and Ac-CoA production in a real-time kinetic substrate assay and endpoint LC–MS detection of Ac-CoA production, respectively (Figure 4A–C). These experiments together showed that MGO had the ability to inhibit ACAT1 activity in vitro and potentially the propensity to do so in cells. CoA does contain a free thiol, which could also interact with MGO and contribute to its inhibition of ACAT1 activity. However, it is probable that CoA with a p*K*_a_ of 9.8^28^ would be far less reactive toward MGO than the ACAT1 cysteine where the catalytic dyad of its active site cysteine could lead to a thiol with a p*K*_a_ as low as three.^29^ Particularly in the cellular environment, where there is an abundance of glutathione with a thiol p*K*_a_ of 8.6,^30^ it is unlikely that CoA would be appreciably modified by MGO or that MGO modification of CoA would be a driving factor behind inhibition of ACAT1 activity.

Another target protein highlighted across our data sets was the central glycolytic enzyme, GADPH, which connects upper and lower glycolysis through metabolism of d-glyceraldehyde phosphate (GAP). MGO modulation of GAPDH activity has been demonstrated in the literature, albeit without a confirmed molecular mechanism,^31,32^ and GAPDH catalysis relies on an active cysteine–histidine dyad comprised of C152 & H179 similar to ACAT1 (Figure S6A). Intriguingly, significant MGO competition was observed at a 37 kDa band matching GAPDH in our in vitro MGO profiling experiments (Figure 1C,D), and MGO competition at C152 in GAPDH was observed in our proteomic profiling studies (Datasets 1–3). Pulse-chase treatment of recombinant GAPDH with MGO followed by IA-alkyne confirmed that reversible modification of one or more cysteines in GAPDH does occur in vitro, and this coincides with reduced GAPDH activity in enzymatic assays (Figure S6B,C).

### MGO Promotes Metabolic Switching by Targeting Key Metabolic Enzymes

ACAT1 catalyzes the cleavage of acetoacetyl-co-enzyme A (AcAc-CoA) to produce two acetyl-CoA (Ac-CoA) groups in conjunction with the consumption of a free CoA molecule^33^ (Figure 4A). In cells, AcAc-CoA can be produced by β-oxidation of even chain fatty acids^34^ and is utilized primarily by one of two pathways. The first is the conversion of AcAc-CoA to two Ac-CoA molecules. The second is the utilization of AcAc-CoA and a molecule of Ac-CoA by HMG-CoA synthase (HGMCS) to produce HMG-CoA, which is subsequently utilized by the mevalonate pathway for cholesterol and isoprenoid biosynthesis^35^ (Figure 4A). We hypothesized that reversible inhibition of ACAT1 by MGO could cause prioritized conversion of AcAc-CoA into the mevalonate pathway. Likewise, GAPDH regulates the flow of the triosephosphates GAP and DHAP—both precursors to MGO—into lower glycolysis. To determine whether the observed MGO regulation of functional cysteines in ACAT1 and/or GAPDH impacts metabolism in cells, we performed kinetic LC–MS analyses of metabolites in these connected pathways following cellular treatment with MGO under conditions that mirrored in situ chemoproteomic profiling experiments.

First, we observed a significant increase in the levels of upper glycolytic metabolites following MGO treatment of HeLa cells, which could be consistent with localized MGO inhibition of GAPDH and perhaps central/lower glycolytic enzymes. In particular, the substrate of GAPDH, glyceraldehyde-3-phosphate (GAP), was increased more than fourfold at the 2 h mark following MGO treatment (Figures 4D and S8B,C and Table S2). To corroborate the potential regulation of glycolysis by MGO in cells, we measured the effect of MGO treatments on bulk metabolic fluxes using Seahorse profiling in HeLa cells. MGO treatment led to dose-dependent reduction in extracellular acidification rate (ECAR)—a measure of glycolytic flux—consistent with steady state metabolomics that show buildup of central glycolytic metabolites around GAPDH (Figure S7).

We also observed significant changes in mevalonate pathway metabolites mevalonate-5-phosphate (MVAP) and mevalonate-5-pyrophosphate (MVAPP) following MGO treatment (Figure 4E,F). The 3–4-fold accumulation of MVAP and MVAPP observed upon MGO treatment is consistent with MGO inhibition of ACAT1, resulting in increased utilization of AcAc-CoA by HMGCS toward mevalonate pathway biosynthesis. We speculate that steady state levels of MVAP and MVAPP are particularly increased due to the fact that mevalonate-5-pyrophosphate decarboxylase, the enzyme that converts MVAPP to isopentenyl diphosphate, is a rate-limiting step of the mevalonate pathway.^36^ To confirm that this mechanism occurs under physiologically relevant MGO concentrations and kinetics, we treated cells with *S*-*p*-bromobenzylglutathione cyclopentyl diester (BBGC), a small molecule inhibitor of GLO1 that will increase endogenous MGO. As with exogenous MGO treatment, we observed significant and time-dependent increases in MVAP and MVAPP levels upon BBGC treatment (Figure 4G,H). These experiments together suggest that MGO regulation of ACAT1 does redirect metabolic flux into the mevalonate pathway though it is conceivable that there are other contributing factors that have yet to be elucidated. Intriguingly, we did not observe significant accumulation of glycolytic metabolites after BBGC treatment (Figure S8D–F), suggesting that the MGO-mediated effects on glycolytic flux may result from a combination of partial inhibition of GAPDH and one or more adjacent glycolytic enzymes by MGO, as has been previously observed by coordinated acetylation and phosphoglyceration.^37,38^ Notably, BBGC inhibition of GLO1 will result in increased endogenous MGO but reduced levels of lactoyl-glutathione, which has been shown to cause acylation of lysine residues within glycolytic enzymes and may contribute to disparate effects of MGO vs BBGC on glycolytic metabolite levels (Figure S8A). Collectively, these results confirm that MGO-mediated modifications impact metabolite flow through key pathways likely through the action of multiple interconnected modifications as in the case of glycolysis and through reversible cysteine modulation observed for ACAT1 and mevalonate pathway metabolism.

### Discussion

In this study, we generated the first proteome-wide map of MGO-dependent cysteine modification both in vitro and in situ. Reactive cysteines were enriched for similar groups of proteins and structural elements in both data sets. These data demonstrated that structural and steric considerations played a larger role than expected in defining the overall landscape of MGO reactivity toward cysteine residues. We anticipate that this insight into reactivity trends and discovery of specific sites of cysteine regulation by MGO will aid in future studies of the proteins and pathways that sense and respond to altered metabolic and redox states in cells.^39^

While this represents a deep look into the reactive profile of MGO, there are clear limitations present in our data set, which likely extend to future attempts to detect reversible metabolite modifications in cells. First, there are known redox-regulated proteins and specific sites that are not observed in our profiles, including sensor residues in KEAP1 and PRDX1/2. The reliance upon lysate-based labeling with IA-alkyne requires interrogation of the proteome in a nonnative, oxidizing environment, and therefore there are likely many sites that could interact with MGO that are not observed using this approach. Second, the dynamic reversibility of cysteine-MGO engagement combined with our use of an irreversible chemical probe to report on cysteine engagement (IA-alkyne) is likely to miss some rapidly reversible modifications. This prompted us to use relatively high MGO doses and short labeling time points. For some reactive cysteine hotspots, like the active site residues in ACAT1, the presence of multiple, proximal nucleophiles may lead to more stable reversible adducts that render them more detectable in this format (Figure 3E). These sites may represent a kinetic middle ground between rapidly reversible sites and irreversible dinucleophile adducts like MICA modifications.^4,13^ Additionally, reversible covalent inhibitors are showing promise as chemical probes^40^ and recently approved drugs, such as the SARS-CoV-2 protease inhibitor Nirmatrelvir.^41^ Expanded efforts to map the interaction landscape of reversible modifications are thus warranted.

We showed that this profiling approach was able to identify functional regulation of cysteine residues, notably the catalytic residues of metabolic enzyme ACAT1. MGO inhibited the activity of ACAT1 in vitro and altered metabolite levels in cells in a manner consistent with inhibition of ACAT1, as well as glycolytic enzyme GAPDH. The altered utilization of AcAc-CoA produced by β-oxidation that is induced by MGO inhibition of ACAT1 represents a potentially important regulatory mechanism. Under conditions of high glycolytic flux during which higher levels of MGO might accumulate, there is likely already sufficient Ac-CoA being produced to fuel the TCA cycle. Thus, it may be beneficial to instead utilize the AcAc-CoA for biosynthesis. Conversely, when there are lower levels of glycolysis and therefore less MGO, the increased ACAT1 activity provides additional Ac-CoA from β-oxidation to sustain the TCA cycle. Collectively, the dynamic and reversible modification of several key cysteines connecting glycolysis, fatty acid oxidation, mevalonate synthesis, and the TCA cycle represents a concerted mechanism by which cells might tune metabolism in response to relative availabilities of key substrates. These and other regulatory sites could be markers of or contributors to diseases characterized by dysregulated glycolysis, such as diabetes and cancer. Further characterization of this and other MGO responsive pathways represent potential opportunities for therapeutic interventions that leverage the increased reliance of many cancers on glycolysis.

## Materials and Methods

All reagents were from Sigma Aldrich, and all bulk solvents were from Thermo Fisher Scientific unless otherwise stated.

### Cell Culture

HEK293, HeLa, and HCT116 cells were purchased from ATCC and were propagated in RPMI 1640 with 2 mM glutamine supplemented with 10% fetal bovine serum and 1% penicillin/streptomycin (Gibco). SILAC labeling was performed by growing cells for at least five passages in lysine and arginine free RPMI 1640 Media for SILAC with 2 mM glutamine supplemented with 10% dialyzed fetal bovine serum and 1% penicillin/streptomycin (Gibco). “Light” and “heavy” media were supplemented with natural lysine and arginine (0.1 mg/mL) and equimolar ^13^C,^15^N-labeled lysine and arginine, respectively.

### IA-Alkyne In Vitro Competition Samples

HeLa, HEK293, or HCT116 cells were washed with PBS buffer collected by scraping in PBS buffer, resuspended in PBS buffer containing EDTA-free complete protease inhibitors (Roche), and sonicated (Fisher Scientific FB-505) for 15 s (30% amplitude, 1 s on, and 1 s off). Insoluble debris was cleared by a 15-min centrifugation at 16,000*g* and 4 °C. Protein concentrations were determined by Bradford assay, and samples were diluted to 2 mg/mL with PBS buffer containing EDTA-free complete protease inhibitors.

For IA-alkyne kinetic competition in Figure 1C, HeLa lysate samples were treated for 1 h with 0, 0.25, or 1 mM MGO at 37 °C followed by treatment with 100 μM IA-alkyne (10 mM stock in DMSO) at RT in the dark for 5, 10, 20, or 30 min. 5 mM iodoacetamide (0.5 M stock) was added, and the samples were incubated for an additional 30 min in the dark.

For IA-alkyne dose–response competition in Figure 1D, HeLa lysate samples were treated for 2 h with 0, 0.05, 0.25, 0.5, or 1 mM MGO at 37 °C followed by 1-h treatment with 0, 1, 10, 50, or 200 μM of IA-alkyne (10 mM stock in DMSO) for untreated lysates and 100 μM IA-alkyne for MGO dose–response competition samples at RT in the dark.

For SILAC proteomics, 1 mM MGO treatment or vehicle was added to 1 mL of 2 mg/mL paired SILAC samples, with half the paired samples receiving MGO treatment on the “light” samples and half receiving MGO treatment on the “heavy” samples. 100 μM IA-alkyne was used for both conditions in each paired sample.

### Rhodamine Labeling and Imaging

For in-gel fluorescence readout, 50 μL of MGO- and IA-alkyne-treated lysate were reacted with 1 μL of 50 mM CuSO_4_, 1 μL of 50 mM TCEP-HCl, 3 μL of 1.67 mM TBTA in 4:1 *t*-butanol/DMSO, and 1 μL of rhodamine azide (1 mM stock in DMSO) at RT for 1 h. The samples were diluted into 4× Laemmli buffer containing 100 mM βME. Samples were prepared for SDS-PAGE by heating to 95 °C for 5 min, cooled to RT, resolved on a 10% SDS-PAGE gel, and imaged on a BioRad ChemiDoc MP Imager.

### IA-Alkyne In Situ Competition Samples

SILAC HeLa cells were grown to confluence in 15 cm plates. Paired SILAC plates were treated with 8 mL of 2 mM MGO or vehicle in corresponding SILAC RPMI for 2 h, with half the paired samples receiving MGO treatment on the “light” samples and half receiving MGO treatment on the “heavy” samples. Cells were washed with PBS buffer collected by scraping in PBS buffer, resuspended in PBS buffer containing EDTA-free complete protease inhibitors (Roche), and sonicated (Fisher Scientific FB-505) for 15 s (30% amplitude, 1 s on, and 1 s off). Insoluble debris was cleared by a 15-min centrifugation at 16,000*g* and 4 °C. Protein concentrations were determined by Bradford assay, samples were diluted to 2 mg/mL with PBS buffer containing EDTA-free complete protease inhibitors, and 1 mL of each sample was kept. Samples were then treated with 100 μM IA-alkyne at RT in the dark for 1 h.

### Sample Processing for Site of Labeling IA-Alkyne Proteomics

Following IA-alkyne labeling, 1 mL of lysate from each condition was reacted with 20 μL of 50 mM CuSO_4_, 20 μL of 50 mM TCEP-HCl, 60 μL of 1.67 mM TBTA in 4:1 *t*-butanol/DMSO, and 10 μL of diazo biotin azide (Click Chemistry Tools and 10 mM stock in DMSO) at RT for 1 h. Corresponding “light” and “heavy” samples were pooled and proteins precipitated via CHCl_3_/MeOH precipitation. Briefly, pooled samples were combined with 2 mL of H_2_O, 4 mL of MeOH, and 1 mL of CHCl_3_ and centrifuged at 3000*g* for 20 min, yielding a protein pellet suspended between solvent layers. Both solvent layers were removed, the pellet was resuspended in MeOH, the samples were centrifuged for 5 min at 9000*g*, and the supernatant was removed. Protein pellets were then resuspended in 1 mL of 8 M urea in PBS. 10 μL of 1 M dithiothreitol (DTT) was added and the samples were heated at 65 °C for 15 min. Samples were allowed to cool to RT. 80 μL of 0.5 M iodoacetamide was added and the samples were incubated for 30 min at RT in the dark. Samples were diluted to a final concentration of 1 M urea with addition of PBS, and 100 μL of prewashed streptavidin agarose beads (Thermo Fisher Scientific) was added and then incubated with end over end rotation at RT for 2 h. The streptavidin beads were washed five times with 10 mL of 1 M urea in PBS and then resuspended in 200 μL of 2 M urea in 25 mM NH_4_HCO_3_. Samples were supplemented with 1 mM MgCl_2_ using a 100 mM stock solution in water and then subjected to trypsin-mediated proteolysis with 2 μg of sequencing grade trypsin (Thermo Fisher Scientific) for 16 h at 37 °C. The streptavidin beads were separated from the supernatant via Micro Bio-Spin column (BioRad) and washed 10 times with 300 μL of PBS. The beads were resuspended in 200 μL of 50 mM Na_2_S_2_O_4_ in PBS and incubated at RT for 1 h to elute the bound site of labeling peptides. The supernatant was collected using Micro Bio-Spin column, and the process was repeated twice. The peptides were desalted using 100 μL Pierce C18 tips (Thermo Fisher Scientific) or self-packed stage tips with C18 SPE (Sigma Aldrich) according to manufacturer’s protocol and then dried via lyophilizer.

### LC–MS/MS Analysis Proteomics

LC–MS/MS analysis for HeLa proteomics samples was performed with an Easy-nLC 1000 ultra-high-pressure LC system (Thermo Fisher Scientific) using a PepMap RSLC C18 column (75 μm × 15 cm; 2 μm, 100 Å, Thermo Fisher Scientific) heated to 45 °C. The LC system was coupled to a Q Exactive HF orbitrap and Easy-Spray nanosource (Thermo Fisher Scientific). Mobile phase A was composed of H_2_O supplemented with 0.1% formic acid, and mobile phase B was composed CH_3_CN supplemented with 0.1% formic acid. The instrument was run at 0.3 μL/min with the following gradient: 2% Buffer B (0–5 min); 2–5% Buffer B (5–6 min); 5–30% Buffer B (6–246 min); 30–90% Buffer B (246–247 min); 90% Buffer B (247–257 min); and 90–2% Buffer B (257–260 min). MS/MS spectra were collected from 0 to 260 min using a data-dependent, top-10 ion setting with the following details: full MS scans were acquired at a resolution of 120,000, scan range of 300–1650 *m*/*z*, maximum IT of 20 ms, AGC target of 3e6, and data collection in profile mode. MS2 scans were performed by HCD fragmentation with a resolution of 60,000, AGC target of 1e5, maximum IT of 120 ms, NCE of 27, and data collection in centroid mode. The isolation window for precursor ions was set to 1.5 *m*/*z*. Peptides with a charge state of 1, 6–8, and unassigned were excluded, and dynamic exclusion was set to 20 s. The S-lens RF level was set to 60 with a spray voltage value of 2.60 kV and an ionization chamber temperature of 300 °C.

LC–MS/MS analysis for HCT116 and HEK293 proteomics samples was performed with an UltiMate 3000 RSLCnano System (Thermo Fisher Scientific) using an Acclaim PepMap RSLC C18 column (75 μm × 15 cm; 2 μm, 100 Å, Thermo Fisher Scientific) with an in-line Acclaim PepMap 100 C18 trap column (75 μm × 2 cm; 3 μm, 100 Å, Thermo Fisher Scientific) heated to 45 °C. The LC system was coupled to an Exploris 480 orbitrap and Nanospray Flex Ion Source with stainless steel emitter tip (Thermo Fisher Scientific). Mobile phase A was composed of H_2_O supplemented with 0.1% formic acid, and mobile phase B was composed of CH_3_CN supplemented with 0.1% formic acid. The instrument was run at 0.3 μL/min with the following gradient: 2% Buffer B (0–5 min); 2–20% Buffer B (5–155 min); 20–32% Buffer B (155–185 min); 32–95% Buffer B (185–186 min); 95% Buffer B (186–190 min); 95–2% Buffer B (190–191 min); and 2% Buffer B (191–200 min). MS/MS spectra were collected from 0 to 200 min using a data-dependent, 2-s cycle time setting with the following details: full MS scans were acquired at a resolution of 120,000, scan range of 300–1650 *m*/*z*, maximum IT of 40 ms, normalized AGC target of 300%, and data collection in profile mode. MS2 scans were performed by HCD fragmentation with a resolution of 60,000, normalized AGC target of 100%, maximum IT of 120 ms, HCD collision energy 30%, and data collection in centroid mode. The isolation window for precursor ions was set to 1.6 *m*/*z*. Peptides with a charge state of 1, 6+, and unassigned were excluded, and dynamic exclusion was set to 40 s. The RF lens % was set to 40 with a spray voltage value of 2.0 kV and an ionization chamber temperature of 300 °C.

Data was processed using the Sequest HT search engine node within the Proteome Discoverer 2.5 software package. Data were searched using a concatenated target/decoy UniProt database of the human proteome. Enzyme specificity was set to trypsin with up to two missed cleavages allowed, and peptide length was set to between 6–144. Precursor mass range was set to 350–6000. Precursor mass tolerance was set to 15 ppm, and fragment mass tolerance was set to 0.02 Da. Up to four dynamic modifications were allowed, including probe-labeled cysteine (+273.1126), heavy lysine (+8.0142), heavy arginine (+10.0083), oxidized methionine (+15.9949), cysteine carboxyamidomethylation (+57.0215), N-terminal acetylation (+42.0106), N-terminal Met-loss (−131.0405), and N-terminal Met-loss + acetylation (−89.0299). A minimum of one peptide was required for protein identification, and false discovery rate was determined using Percolator with FDR rate set at 1%. For localization of modifications, a score of 75% or greater was required.

Prior to quantification, chromatographic alignment was performed with a maximum retention time difference of 10 min allowed and a minimum signal/noise threshold of five required for feature mapping. SILAC ratios were determined using precursor-based quantification in a pairwise manner based on peak area without normalization or scaling using a maximum ratio of 40. Peptides containing the same site of probe modification were grouped, and aggregate statistics were calculated at the site of modification level for each unique site of modification within a data set. Data presented are representative of four independent biological experiments, and sites of modification were required to be quantifiable at least twice across the biological replicates. Sites for which only singletons were detected and had singletons detected in both MGO and vehicle-treated samples were discarded as unreliably quantifiable.

### Recombinant Protein IA vs MGO

50 μg/mL recombinant ACAT1 or GAPDH (human, Sigma Aldrich) in PBS was incubated with the indicated concentrations of MGO for 2 h at 37 °C followed by 30-min treatment with 50 μM IA-alkyne at RT in the dark. 1 mM iodoacetamide was added, and the samples were incubated for an additional 30 min in the dark. Click chemistry and imaging with rhodamine azide was then performed as described above.

For washout experiments, the ACAT1 was buffer exchanged into fresh PBS following MGO treatment using an Amicon ultra centrifugal filter unit with 10 kDa cutoff (Millipore), after which IA-alkyne labeling and rhodamine azide labeling were performed as described above.

### ACAT1 In Vitro Assay

Recombinant ACAT1 (200 ng/mL in PBS) or vehicle was incubated with 2 mM MGO or vehicle for 2 h at 37 °C. 10 mM aqueous stocks of coenzyme A trilithium salt (CoA, Sigma Aldrich) and acetoacetyl coenzyme A (AcAc-CoA, Cayman Chemicals) were added to 40 mM MgCl_2_ in PBS to give 400 μM final concentration of each substrate. The substrate mixture was allowed to preequilibrate at 37 °C, then mixed in a 1:1 ratio with the reaction solutions, and imaged at 303 nm^33^ once a minute using a Synergy Neo HTS Microplate Reader (BioTek). Relative AcAc-CoA consumption for each condition was calculated by the normalized absolute difference between the 303 nm absorbance in the ACAT1 containing reactions and the corresponding ACAT1-less control reactions.

For MS/MS monitoring of Ac-CoA production, aliquots of the reactions were taken at the indicated time points and mixed with MeOH in a 4:1 MeOH/sample ratio to quench the enzymatic reaction. Internal deuterated standards, 1 μL of 10 mM d3-serine, were added to the extraction solution for sample normalization, the samples were centrifuged for 15 min at 16,000*g*, and the supernatant was kept for immediate LC–MS/MS analysis.

### GAPDH In Vitro Assay

Recombinant GAPDH (50 μg/mL in PBS) was incubated with 2 mM MGO or vehicle for 1 h at 37 °C and then added 1:1 vol/vol with PBS solution containing NAD+ (Sigma Aldrich) and d/l-glyceraldehyde-3-phosphate (GAP, Sigma Aldrich) to give a final concentration of 0.4 mM NAD+ and 2 mM GAP. Absorbance was measured at 340 nm once a minute to monitor NADH production.

### Metabolomics of MGO and BBGC Treatment in Cells

For profiling of MGO adduct formation in Figure S3, two million HeLa cells were plated in 10 cm plates and were allowed to grow for 24 h before treatment. Cells were treated in 5 mL of RPMI with 1 mM MGO for 0, 1, 2, 4, or 8 h.

For profiling of polar metabolite changes in response to MGO or BBGC treatment in Figures 4 and S8, two million HeLa cells were plated in 10 cm plates and were allowed to grow for 24 h before treatment. Cells were treated in 5 mL of RPMI with 2 mM MGO for 0, 0.5, 1, 2, or 4 h or with 20 μM BBGC for 0, 2, 4, or 8 h.

Cells were collected by trypsinization, washed once with PBS, and resuspended in 300 μL of an 80:20 mixture of MeOH/H_2_O. Internal deuterated standards, 1 μL of 10 mM d3-serine, were added to the extraction solution for sample normalization. The mixture was sonicated (Fisher Scientific FB-505) for 10 s (30% amplitude, 1 s on, and 1 s off) followed by a 10-min centrifugation at 16,000*g* and 4 °C. The supernatant was collected and dried using SpeedVac.

### LC–MS/MS Analysis of Metabolites

Dried metabolome samples for MGO adduct analysis were resuspended in 50 μL of 0.1% TFA in H_2_O and clarified by centrifugation at 16,000*g* for 10 min. Extracellular samples were processed similarly but in a volume of 200 μL. Targeted MS/MS analyses were performed on an Agilent triple quadrupole LC–MS/MS instrument (Agilent Technologies 6460 QQQ) set to positive ion mode. The capillary voltage was set to 4.0 kV. The drying gas temperature was 300 °C, flow rate = 5 L/min, and nebulizer pressure = 45 psi. The mass spectrometer was run in MRM mode with delta EMV(+) set to 200. MRM parameters are listed in extended data table 1. Chromatography was performed with a Phenomenex Gemini C18 column (50 × 4.6 mm, 5 μm) at a flow rate of 0.4 mL/min. Mobile phase A was composed of H_2_O supplemented with 0.1% TFA, and mobile phase B was composed of CH_3_CN supplemented with 0.1% TFA. The instrument was run at 0.4 mL/min with the following gradient: 0% Buffer B (0–3 min); 0–100% Buffer B (3–10 min); 100% Buffer B (10–11 min); 100–0% Buffer B (11–12 min); and 0% Buffer B (12–15 min). Relative metabolite abundance was quantified by integrated peak area for the given MRM-transition normalized to that of the internal standard.

Dried metabolome samples for polar metabolite analysis were resuspended in 50 μL of 80:20 MeOH/H_2_O and centrifuged at 16,000 g for 10 min, and the supernatant was kept. Targeted MS/MS analysis was performed on an Agilent triple quadrupole LC–MS/MS instrument (Agilent Technologies 6460 QQQ) set to negative ion mode. The capillary voltage was set to 3.5 kV. The drying gas temperature was 300 °C, the drying gas flow rate was 5 L/min, and the nebulizer pressure was 45 psi. The mass spectrometer was run in MRM mode with delta EMV(−) set to 0. MRM parameters are listed in extended data table 1. Hydrophilic interaction chromatography was performed using a Phenomenex Luna-NH_2_ column (50 × 4.6 mm, 5 μM). Mobile phase A was composed of CH_3_CN supplemented with 0.2% NH_4_OH, and mobile phase B was composed of 95/5 H_2_O/CH_3_CN supplemented with 50 mM NH_4_OAc and 0.2% NH_4_OH. The instrument was run at 0.4 mL/min with the following gradient: 0% Buffer B (0–2 min); 0–100% Buffer B (2–15 min); 100% Buffer B (15–20 min); 100–0% Buffer B (20–21 min); and 0% Buffer B (21–23 min). Injection volume was 15 μL for all samples. Relative metabolite abundance was quantified by integrating the peak area for the given MRM-transition and normalizing to that of the internal d3-serine standard. Data presented are representative of four independent biological experiments each containing two technical replicates for a given condition. Heat maps for metabolomics data were generated using Morpheus (https://software.broadinstitute.org/morpheus/).

### Seahorse Assays

ECARs were analyzed with a Seahorse 96XF Analyzer (Seahorse Bioscience) using the XF Glycolysis Stress Test Kit (Seahorse Bioscience) according to the manufacturer’s protocol with slight modification. Briefly, 20,000 HeLa cells per well were plated on a 96-well Seahorse microplate in RPMI 24 h prior to the experiment. The medium was replaced 4 h before the experiment with Seahorse XF Base Medium supplemented with 0, 0.25, or 1 mM MGO. Cells were then incubated at 37 °C (low CO_2_) for 4 h. The day prior to the experiment, the Seahorse cartridge was hydrated with water overnight at 37 °C (low CO_2_). Water was then replaced with calibrating solution (Seahorse Bioscience) 2 h prior to the experiment followed by loading the cartridge with glucose—PORT A (10 mM), oligomycin (3 μM)—PORT B, and 2-deoxyglucose (50 mM)—PORT C. All three additives were dissolved in Seahorse XF Base Medium supplemented with MGO matched to the concentrations of MGO added to the cells in the corresponding positions on the 96-well microplate. Glycolytic parameters were calculated according to manufacturer’s protocols. ECAR plots presented are representative of three independent biological experiments each containing six technical replicates per condition.

## Data Availability

Raw proteomics data are deposited in the Proteome Xchange Consortium through MassIVE under accession number MSV000090948.

## Supporting Information Available

Figures S1–S5: supplementary structures and additional data related to IA-alkyne profiling of cysteine modification by MGO. Figures S6–S8: supplementary data related to MGO regulation of GAPDH function and glycolysis. Tables S1 and S2: MS/MS parameters for metabolomics (PDF)
Data sets 1–4: proteomics data for IA-alkyne profiling experiments (XLSX)
