# Supplementary material for: Widespread, Reversible Cysteine Modification by Methylglyoxal Regulates Metabolic Enzyme Function
Source: ACS Chem Biol. 2022 Dec 23;18(1):91–101. doi: 10.1021/acschembio.2c00727 (PMC9872086; doi:10.1021/acschembio.2c00727)
Supplement: Supplementary file 1 — cb2c00727_si_001.pdf [file cb2c00727_si_001.pdf]

Supporting Information for:

Widespread, Reversible Cysteine Modification by Methylglyoxal Regulates Metabolic Enzyme Function

John S. Coukos, Chris W. Lee, Kavya S. Pillai, Kimberly J. Liu & Raymond E. Moellering*
Department of Chemistry, The University of Chicago
929 E. 57th Street, Chicago, IL 60637, United States

*e-mail correspondence to: rmoellering@uchicago.edu

Supplemental Materials and Methods

All reagents were from Sigma-Aldrich, and all bulk solvents were from Thermo Fisher Scientific unless otherwise stated.

IA-alkyne synthesis. Iodoacetic anhydride (100 mg, 0.28 mmol) was dissolved in anhydrous CH_3CN (10 mL). Propargylamine (17.9 µL, 0.28 mmol) and Et_3N (97µL, 0.7 mmol) were added and the reaction stirred in the dark at room temperature (RT) for 2 hours. The reaction was partitioned between ethyl acetate (10 mL) and H_2O (10 mL) and then extracted one additional time with ethyl acetate. The organic layers were combined, dried over Na_2SO_4, concentrated in vacuo, and then purified by silica column yielding a pale yellow solid (28 mg, 36%): ^1H NMR (DMSO-d_6, 400 MHz) δ 8.67 (s, 1H), 3.86 (m, 2H), 3.64 (d, 2H, J = 4.0 Hz), 3.16 (dt, 1H, J = 2.8, 2.8).

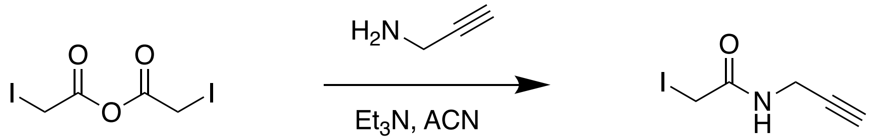

Methylglyoxal synthesis and quantification. High purity MGO was prepared by acidic hydrolysis of MG-1,1-dimethylacetal followed by fractional distillation and concentration quantified by colorimetric assay[33]. Briefly, 6 mL of MG-1,1-dimethylacetal was added to 100 mL of 2.5% (v/v) sulfuric acid and refluxed for 1 hour. The product was purified by fractional distillation under reduced pressure. The first fraction collected was discarded due to methanol impurity. To quantify MGO concentration, aliquots of the fractions were diluted with 50 mM sodium phosphate buffer pH 7.4 to be below an estimated 2 mM concentration and reacted with equal volume of 40 mM aminoguanidine in phosphate buffer for 5-6 hours at 37°C. Absorbance was measured at 320 nm and compared to a calibration curve generated by serial dilutions of 3-amino-1,2,4-triazine in phosphate buffer. MGO fractions were then diluted to 50 mM stock solutions using phosphate buffer and pH was confirmed to be 7.4. MGO stocks were stored at -80°C until use.

Structural and motif analyses. Secondary structure, solvent accessibility and protein disorder were predicted using RaptorX (http://raptorx.uchicago.edu)[34]. Consensus sequence logos and secondary structure logos were generated using the online pLogo tool (https://plogo.uconn.edu/) as published[35]. Input sequences consisted of the protein sequence within 5 residues of the site of modification, and sites that were under 5 residues away from the N- or C- terminus of the protein were excluded from the analysis.

ACAT1 and GAPDH crystal structure images. Generation of images from crystal structures for ACAT1 and GAPDH was done in PyMOL. Crystal structures were downloaded from the RCSB Protein Data Bank (structure 1U8F for GAPDH[36], and structure 2F2S for ACAT1, unpublished).

ACAT1 expression and purification. N-terminal His_6-tagged ACAT1 plasmid was a gift from Cheryl Arrowsmith (Addgene plasmid #25510; http://n2t.net/addgene:25510 ; RRID:Addgene_25510, unpublished). His_6-ACAT1 was expressed in *Escherichia coli strain* BL2(DE3). Transformed cells were grown in LB broth supplemented with kanamycin to OD_{600} = 0.6 and induced with 1 mM IPTG at 25°C for 16 hours. Cells were harvested and then lysed on ice by sonication (Fisher Scientific FB-505) in buffer 1 (20 mM Tris pH 7.4 and 100 mM NaCl) supplemented with 1 mM PMSF and 0.5 mM DTT for 10 minutes (50% amplitude, 2 seconds on, 5 seconds off). The lysate was centrifuged at 16,000g for 15 minutes at 4°C and the supernatant loaded onto Ni-NTA resin (Qiagen). The resin was washed with buffer 1 supplemented with 40 mM imidazole and then the protein was eluted in buffer 1 supplemented with 250 mM imidazole. Purified protein was dialyzed overnight with buffer 1 and then buffer exchanged into buffer 1 supplemented with 10% glycerol and 1 mM DTT using an Amicon ultra centrifugal filter unit with 10 KDa cutoff (Millipore). Protein was diluted to 1 mg/mL, aliquoted, and stored at -20°C for later use. Immediately before use in biochemical assays, recombinant ACAT1 buffer exchanged into PBS.

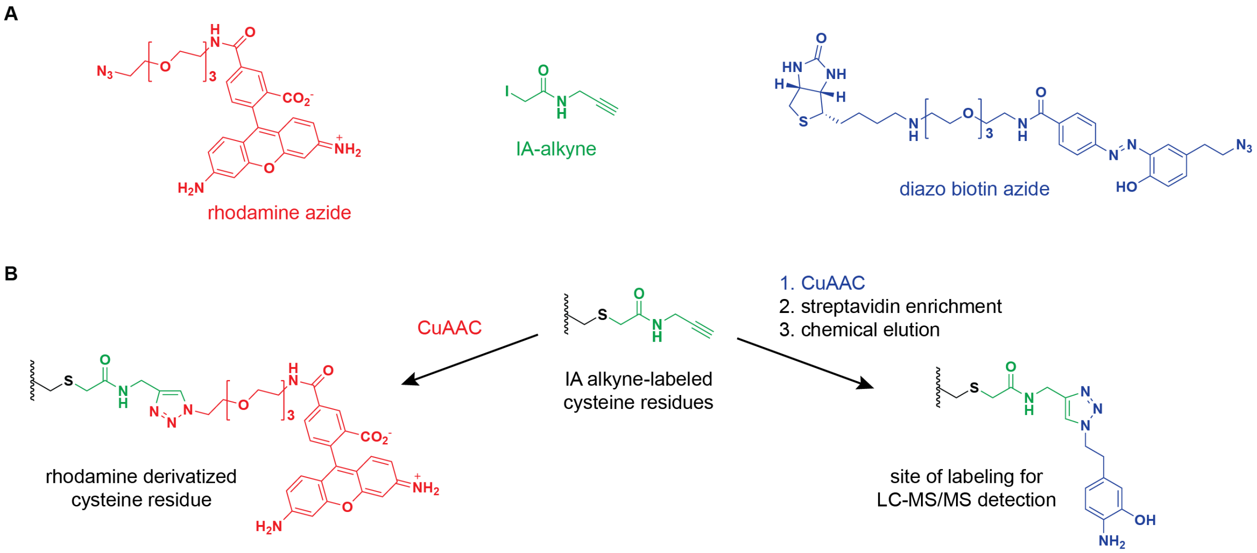

Figure S1 Structures of probes and labeled residues. **(A)** Structures of probes used in this study. **(B)** Structures of cysteine residues labeled with the IA-alkyne probe and derivatized using copper(I) catalyzed alkyne-azide click chemistry (CuAAC).

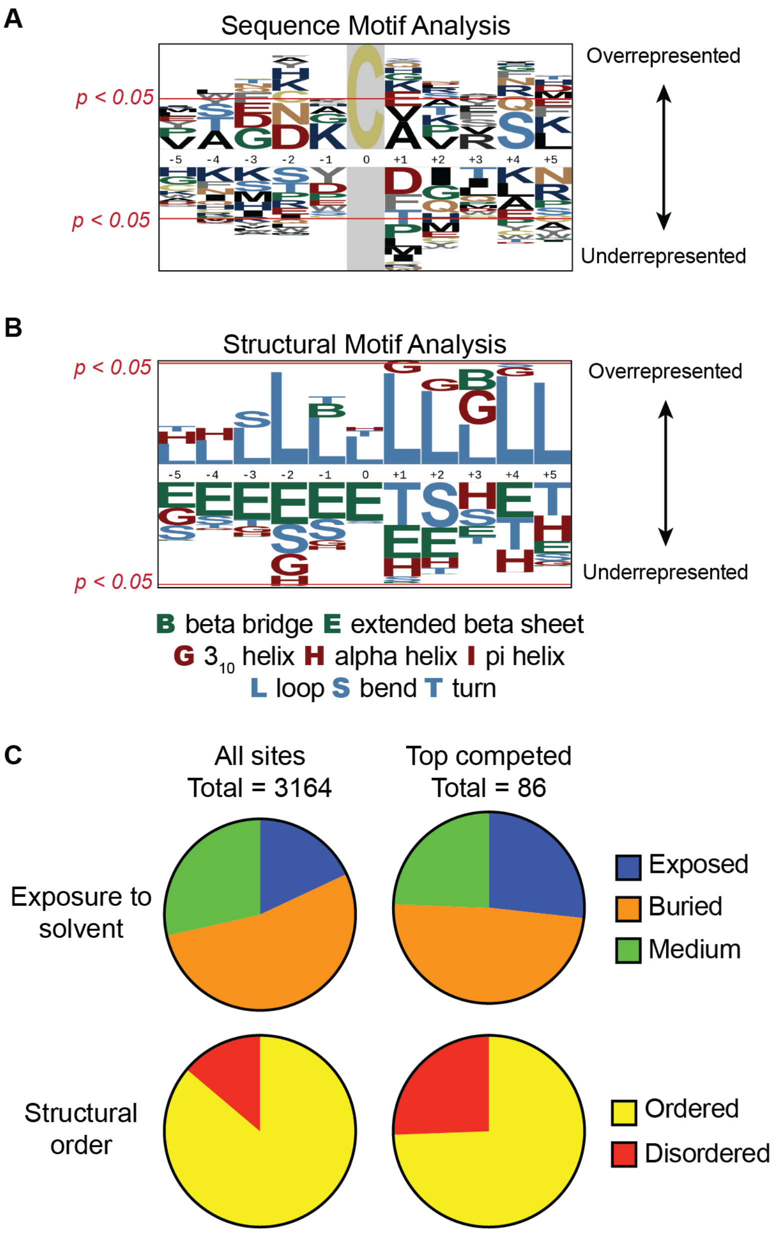

Figure S2 MGO-modified sites are enriched in unstructured, solvent-exposed loop regions. **(A-B)** Sequence (A) and structural motif (B) analysis performed for top-competed modified sites from IA-alkyne SILAC experiments using MGO- or vehicle-treated HeLa, HEK293, and HCT116 cells with a ratio > 2.5 in two or more cell lines (total of 86) with a background consisting of all overlapping modified sites (total of 3164) Red line indicates the cutoff for significant log-odds of the binomial probability of either over- or underrepresentation (p < 0.05). **(C)** Overlapping sites from IA-alkyne SILAC experiments were grouped by level of exposure to solvent (top) and structural order or disorder (bottom). Grouping was performed with all overlapping modified sites (left), as well as the top competed sites (right).

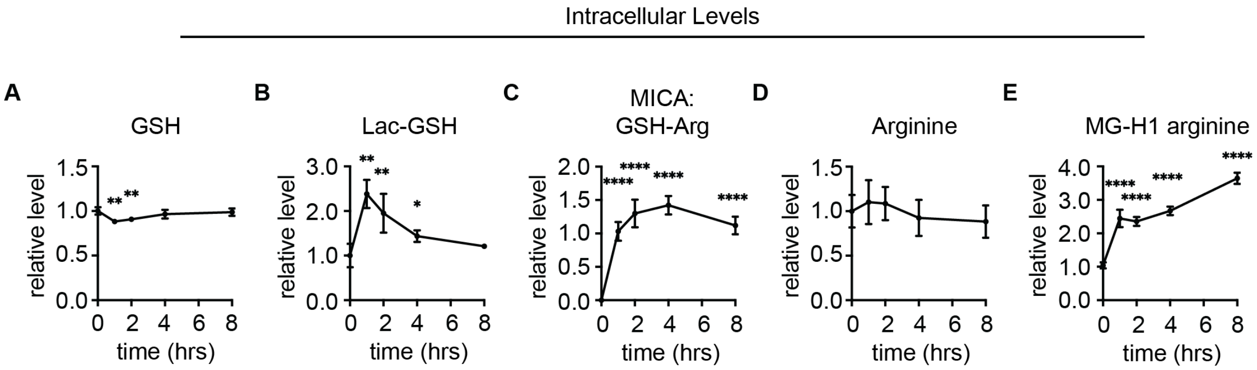

Figure S3 Intracellular kinetics of MGO adduct levels. **(A-E)** Intracellular levels of glutathione (**A,** GSH), lactoyl-glutathione (**B,** Lac-GSH), MICA crosslinks (**C,** GSH-Arg), Arginine (**D**), and MG-H1 Arginine (**E**) in HeLa cells treated with 1 mM MGO for indicated times. Relative LC-MS/MS quantification of each metabolite was determined using the condition at which the maximum MS-transition EIC was detected. Statistical analysis is by two-sided unpaired Student's t-test comparing individual timepoints to their corresponding 0-hour control. *p< 0.05; **p< 0.01; ***p< 0.001; ****p< 0.0001.

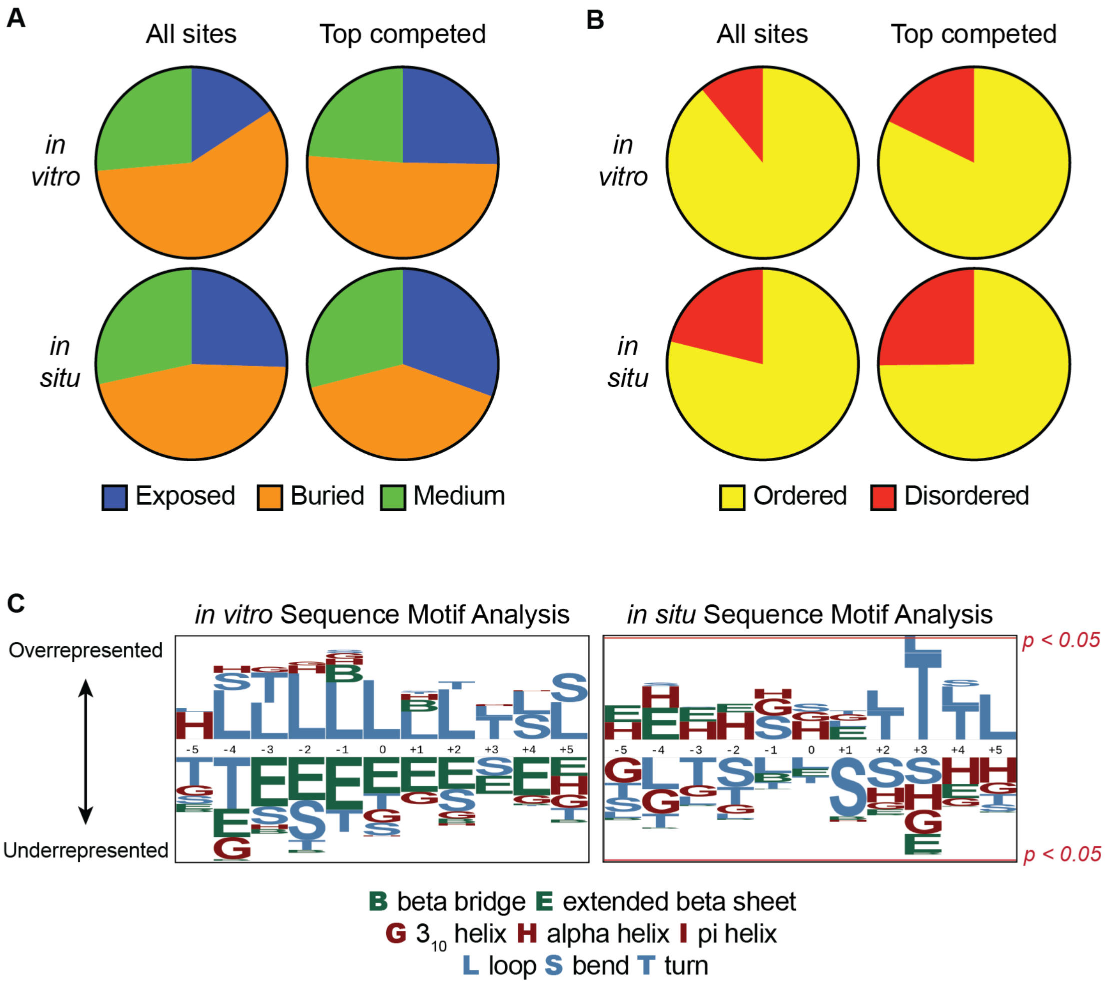

Figure S4 Structural differences surrounding MGO-modified sites in HeLa cell lysate versus whole cells. **(A-B)** Modified sites from IA-alkyne SILAC experiments using MGO- or vehicle-treated HeLa cell lysates (*in vitro*, top) or cells (*in situ*, bottom) were grouped by level of exposure to solvent **(A)** and structural order or disorder **(B)**. Grouping was performed with all identified sites (left; *in vitro* = 2661; *in situ* = 3170),

as well as top competed sites with a ratio > 2.5 (right; *in vitro* = 281; *in situ* = 131). **(C)** Structural motif analysis surrounding modified sites identified *in vitro* (left) and *in situ* (right). Red line indicates the cutoff for significant log-odds of the binomial probability of either over- or underrepresentation of the indicated secondary structures.

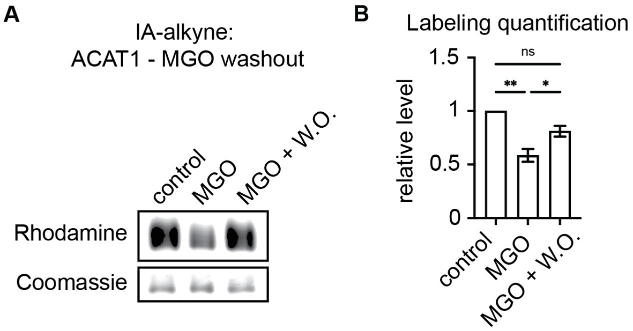

Figure S5 MGO modification of ACAT1 cysteines is reversible. **(A-B)** Representative rhodamine gel **(A)** and quantification of labeling **(B)** of recombinant ACAT1 (0.05 mg/mL) that was treated for 1 hour with vehicle or 2 mM MGO followed by optional buffer exchange wash out and then IA-alkyne treatment for 30 minutes. Data in (B) is mean ± S.E.M from *n* = 3 independent biological replicates. Statistical analysis in (B) is by ordinary one-way analysis of variance (ANOVA). *p< 0.05; **p< 0.01; ***p< 0.001; ****p< 0.0001. W.O., wash out.

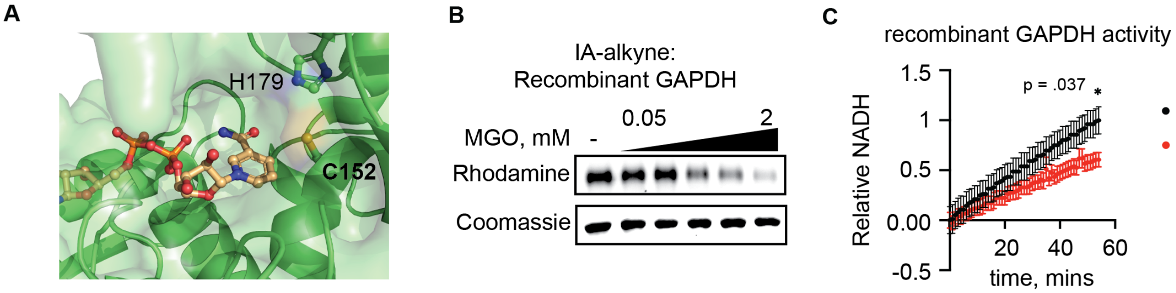

Figure S6 Methylglyoxal inhibits GAPDH in vitro by modifying catalytic residue C152 **(A)** Active site of human GAPDH with C152 and H179 catalytic residues highlighted (PDB Accession: 1U8F). **(B)** Dose-dependent competition of GAPDH and IA-alkyne by MGO *in vitro.* Rhodamine gel of recombinant GADPH (0.05 mg/mL) was pre-treated with indicated concentrations of MGO for 2 hours followed by IA-alkyne treatment. **(C)** Relative production of NADH by recombinant GAPDH treated with MGO (red) or vehicle (black). Data in (C) are mean ± S.E.M from *n* = 8 independent biological replicates. Statistical analysis in (C) is by one-sided unpaired Student's t-test. *p< 0.05; **p< 0.01; ***p< 0.001; ****p< 0.0001.

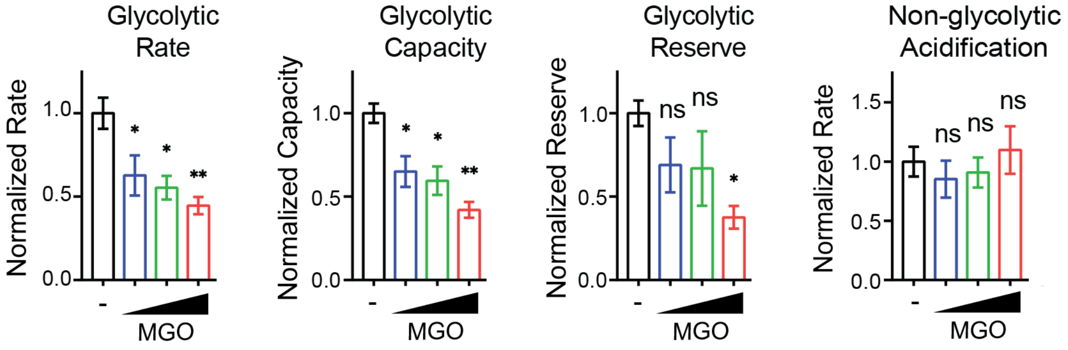

Figure S7 Effect of MGO on bulk glycolytic properties. HeLa cells were treated with MGO (0.25, 0.5, and 1 mM) and glycolytic rate, glycolytic capacity, glycolytic reserve, and non-glycolytic acidification were assessed. Data are mean ± S.E.M from n = 3 biological replicates consisting of

6 technical replicates each. Statistical analyses are by two-sided unpaired Student's t-test comparing individual dose points to their corresponding control. *p< 0.05; **p< 0.01; ***p< 0.001; ****p< 0.0001.

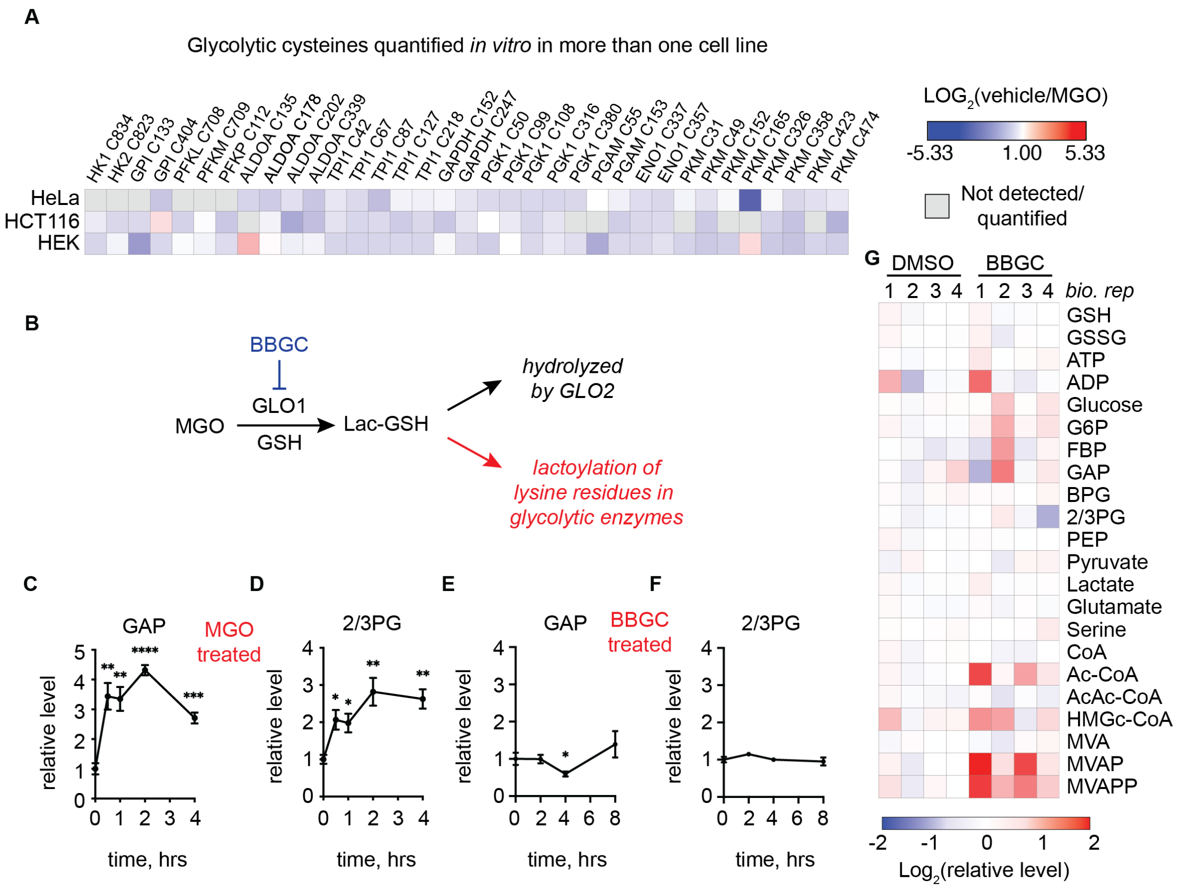

Figure S8. Lactoylation is a possible mechanism for the inhibition of glycolysis by MGO. **(A)** Heatmap depicting the MGO competition ratio of detected cysteines in glycolytic enzymes. **(B)** Schematic depicting the conversion of MGO into lactoyl-glutathione, potential fates of cellular lactoyl-glutathione, and the role of BBGC inhibition in these processes. (**C-D)** LC-MS quantification of glyceraldehyde-3-phosphate (GAP) (B) and 2- and 3-phosphoglycerate (2/3PG) (C) in HeLa cells treated with MGO (2 mM) at the indicated time points. **(E-F)** LC-MS quantification of glyceraldehyde-3-phosphate (GAP) (D) and 2- and 3-phosphoglycerate (2/3PG) (E) in HeLa cells treated with GLO1 inhibitor S-p-bromobenzylglutathione cyclopentyl diester (BBGC, 20 μM) at the indicated time points. **(G)** Representative LC-MS quantification of indicated metabolites in HeLa cells treated with BBGC (20 μM) or DMSO for 8 hours. Data are mean ± S.E.M. from *n* = 4 (B-C) or 8 (D-E) independent biological replicates. Statistical analysis in (C-F) is by two-sided unpaired Student's t-test comparing individual timepoints to their corresponding 0-hour control. *p< 0.05; **p< 0.01; ***p< 0.001; ****p< 0.0001.

Table S1: Acquisition parameters used for negative mode targeted metabolomics measurements.

Metabolite	Precursor ion	MS1 resolution	Product ion	MS2 resolution	Dwell	Fragmentor	Collision energy	Retention time (min)
GSSG	610.7	Wide	305.9	Unit	25	100	15	11.9
ATP	506	Wide	159	Unit	100	100	25	14.6
HMG-CoA	454.6	Wide	79	Unit	100	100	60	14.2

ADP	425.8	Wide	134	Unit	25	100	15	13.1
AcAc-CoA	424.5	Wide	79	Unit	100	100	60	12.9
Ac-CoA	403.5	Wide	79	Unit	25	100	60	13.0
CoA	382.5	Wide	79	Unit	100	100	60	13.6
1,6-FBP	339.1	Wide	96.9	Unit	100	100	20	13.2
MVAPP	306.9	Wide	79	Unit	100	100	23	12.4
GSH	305.7	Wide	143	Unit	25	100	15	10.7
BPG	264.9	Wide	96.9	Unit	5	86	21	22.3
G6P	258.9	Wide	79	Unit	100	100	60	11.2
MVAP	227.03	Wide	79	Unit	100	100	23	12.4
2PG	184.98	Wide	78.9	Unit	25	86	21	12.4
Glucose	179.05	Wide	89.2	Unit	25	68	12	8.2
GAP	169	Wide	96.9	Unit	100	100	5	12.6
PEP	166.97	Wide	79	Unit	25	78	9	12.7
Mevalonate	147.1	Wide	59.1	Unit	100	100	20	12.5
Glu	146.1	Wide	102.1	Unit	25	100	5	10.2
Serine	104.2	Wide	73.8	Unit	100	100	5	9.6
Lactate	89.1	Wide	43	Unit	25	100	20	8.7
Pyruvate	87.1	Wide	43	Unit	25	100	10	12.5
d3-Serine	107.05	Wide	75.1	Unit	25	18	9	9.6

Table S2: Acquisition parameters used for positive mode targeted metabolomics measurements.

Metabolite	Precursor ion	MS1 resolution	Product ion	MS2 resolution	Dwell	Fragmentor	Collision energy	Retention time (min)
Arginine	175. 1	Wide	70.1	Unit	25	106	25	1.7
MG-H1 Arginine	229.1	Wide	70.1	Unit	100	110	29	1.9
Glutathione	308.1	Wide	76.1	Unit	100	106	29	2.4
Lactoyl-Glutathione	380.1	Wide	76.1	Unit	100	106	41	6.0
MICA: GSH-Arg.	518.2	Wide	389.2	Unit	100	192	17	5.8
d_3-Serine	109.07	Wide	63.1	Unit	100	40	13	1.7
